# Albumin–bilirubin score is a useful predictor of worsening liver reserve after stereotactic body radiation therapy in elderly Japanese patients with hepatocellular carcinoma

**DOI:** 10.1093/jrr/rrae006

**Published:** 2024-02-26

**Authors:** Yuki Yoshino, Gen Suzuki, Hiroya Shiomi, Takuya Kimoto, Sho Seri, Hideya Yamazaki, Kei Yamada

**Affiliations:** Department of Radiology, Kyoto Prefectural University of Medicine, 465 Kajii-cho, Kawaramachi-Hirokoji, Kamigyo-ku, Kyoto 602-8566, Japan; Department of Radiology, Kyoto Prefectural University of Medicine, 465 Kajii-cho, Kawaramachi-Hirokoji, Kamigyo-ku, Kyoto 602-8566, Japan; Department of Radiation Oncology, Osaka University, 2-2 Yamadaoka, Suita, Osaka 565-0871, Japan; Department of Radiology, Soseikai CyberKnife Center, Fushimi-ku, Kyoto 612-8248, Japan; Department of Radiology, Kyoto Prefectural University of Medicine, 465 Kajii-cho, Kawaramachi-Hirokoji, Kamigyo-ku, Kyoto 602-8566, Japan; Department of Radiology, Kyoto Prefectural University of Medicine, 465 Kajii-cho, Kawaramachi-Hirokoji, Kamigyo-ku, Kyoto 602-8566, Japan; Department of Radiology, Kyoto Prefectural University of Medicine, 465 Kajii-cho, Kawaramachi-Hirokoji, Kamigyo-ku, Kyoto 602-8566, Japan; Department of Radiology, Kyoto Prefectural University of Medicine, 465 Kajii-cho, Kawaramachi-Hirokoji, Kamigyo-ku, Kyoto 602-8566, Japan

**Keywords:** hepatocellular carcinoma, stereotactic body radiation therapy, albumin–bilirubin score, Child-Pugh score

## Abstract

The prognosis of patients with hepatocellular carcinoma (HCC) is closely related to their liver reserves. The Child-Pugh (CP) score has traditionally been used to evaluate this reserve, with CP Grade B (CP score ≥ 7) associated with a higher risk of radiation-induced liver disease after stereotactic body radiation therapy (SBRT). However, the CP score has limitations, as it does not accurately assess liver reserve capacity. The albumin–bilirubin (ALBI) score has been introduced as a meticulous indicator of liver reserve for the treatment of HCC. We retrospectively evaluated the role of the ALBI score in estimating the worsening liver reserve in 42 patients with HCC treated with SBRT using CyberKnife between 2015 and 2023. The median biologically effective dose (*α*/*β* = 10 Gy) was 100 Gy. For a median follow-up duration of 17.4 months, the 1-year overall survival (OS), local control (LC) and progression-free survival (PFS) rates were 100, 98 and 62%, respectively. Worsening liver reserve was defined as an increase in the modified ALBI grade or CP score within 1 year after SBRT. Univariate and multivariate analyses showed that the baseline ALBI score (≥−2.7 vs <−2.7) was the only significantly different predictor of worsening liver reserve. The OS and LC rates after SBRT for HCC were satisfactory. However, the PFS was poor, and recurrent HCC will require additional treatment. It is clinically important to predict the liver reserve capacity after SBRT, and the baseline ALBI score is a useful predictor.

## INTRODUCTION

Hepatocellular carcinoma (HCC) is the most common malignant liver tumour [1]. As per GLOBOCAN 2020, HCC is the sixth most common cancer globally and the third leading cause of cancer-related mortality [2].

According to clinical practice guidelines, various treatment modalities are available, such as liver transplant, hepatectomy, radiofrequency ablation (RFA), microwave ablation, percutaneous ethanol injection, transarterial chemoembolization (TACE), transarterial radioembolization and radiation therapy [3].

Stereotactic body radiation therapy (SBRT) can be used even if surgery or local puncture therapy for HCC is difficult to perform for medical reasons (e.g. surgery: inoperable; local puncture therapy: target tumour is close to a major blood vessel, bile duct, or diaphragm; target tumour cannot be visualized by ultrasound) [4]. HCC has a good radiation sensitivity [5]. These advantages have recently made SBRT a popular and effective local treatment for HCC [6–9].

It is well known that the prognosis of affected patients depends on the liver reserve [10]. The Child-Pugh (CP) score has long been used to assess the liver reserve capacity. CP Grade B (CP score ≥7) is known to increase the frequency of Grade 3 or higher radiation-induced liver disease (RILD) after SBRT [11–13]. However, CP scores have problems because they are discontinuous variables, subjective and ambiguous in their assessment of encephalopathy and ascites. They are also not a statistically valid method. In addition, CP Grade A cases constitute the majority of SBRT patients, but there are cases of post-RT worsening of liver reserve among patients with CP Grade A. HCC also tends to recur outside the radically treated area [14, 15]. However, SBRT may complicate additional treatments if the liver reserve is impaired. Additional risk factors for post-SBRT worsening of the liver reserve are being sought. The albumin–bilirubin (ALBI) score has emerged as an accurate indicator of liver reserve after HCC treatment [16]. It is a promising tool for assessing liver function, as it is composed of two commonly assessed serum laboratory values. The ALBI score was reported useful in assessing the hepatotoxicity after SBRT for HCC [17–19].

The purpose of this study was to retrospectively evaluate whether the ALBI score is a useful predictor of worsening liver reserve for Japanese patients with HCC treated by SBRT at a Japanese institution, the Soseikai CyberKnife Center. Hepatotoxicity after SBRT is often defined as CP scores of ≥2 [17–21]. However, worsening liver reserve after SBRT in this study was defined as deterioration of the CP score or modified ALBI (mALBI) grade [22]. If the ALBI score is useful for assessing the liver reserve capacity, it should be added to the outcome. In addition, liver reserve is associated with treatment strategy after SBRT regardless of whether CP scores worsened (≥2). Only few studies have been conducted on the usefulness of the ALBI score for hepatotoxicity after SBRT, but none have involved Japanese patients [17–19]. This is the first study to include the ALBI score in assessing liver function deterioration.

## MATERIALS AND METHODS

### Patients

This retrospective study reviewed the data of 42 patients with HCC extracted from the Soseikai CyberKnife Center database. The investigation was conducted from August 2015 to August 2023.

HCC is primarily diagnosed based on imaging studies because pathological confirmation is not feasible in candidates for SBRT. During the follow-up of patients with liver disease, nodules with sizes of ≥1 cm were diagnosed as HCC based on typical hallmarks. These include hypervascularity in the arterial phase and washout in the portal, venous and delayed phases of hypervascular HCC. The imaging techniques included a combination of contrast-enhanced ultrasonography, four-phase multi-detector computed tomography (CT), dynamic contrast-enhanced magnetic resonance imaging (MRI) and CT during hepatic arteriography and arterioportography. Diagnosis was established based on a review of imaging studies [23] and clinical practice guidelines [24]. The clinical stage was determined according to the TNM Classification of Malignant Tumours, eighth edition [25].

The patient and radiotherapy plan characteristics are shown in Table 1. Before SBRT, the CP scores were <7 for 95% and ≥7 for 5% of the patients, and mALBI grades were 1, 2a, 2b and 3 for 50, 19, 29 and 2% of patients, respectively. The liver SBRT was the first treatment for 8/42 cases (19%). Only two patients had a history of liver irradiation before SBRT and 2 years after proton therapy. The hepatic hilum was targeted in 6/42 cases (14%).

**Table 1:** Patient characteristics and treatment detail

Factors	Starta	Number or median (range)	Rate
All patients		42	100%
Age(years old)		79 (58–90)	
Sex	Male	29	69%
	Female	13	31%
Performance status(ECOG)	0	28	67%
	1	13	31%
	2	1	2%
Baseline CP score	< 7	40	95%
	≥7	2	5%
Baseline mALBI grade	1	21	50%
	2a	8	19%
	2b	12	29%
	3	1	2%
HBV	+	2	5%
	−	39	93%
	Unknown	1	2%
HCV	+	21	50%
	−	20	48%
	Unknown	1	2%
Stage	IA	17	40%
	IB	5	12%
	II	20	48%
SBRT total dose (Gy)	50 Gy/5 fr	25	59%
	40 Gy/4 fr	12	29%
	Other	5	12%
BED-10 (Gy)		100 (60–100)	
PTV (cc)		22 (8.5–95)	
Mean liver dose (Gy)		7.8 (3.7–14.8)	
Normal liver volume (cc)		1102 (620–1598)	

### Treatment

The treatment plan included a Vac-Lok fixation. Abdominal compression was performed. The gross tumour volume was calculated from the CT images acquired during free breathing. The planning target volume (PTV) was set considering the respiratory motion, setup margins and subclinical margins from the 4DCT.

SBRT was performed using a CyberKnife radiosurgery system (Accuray, USA) with 6MV X-rays. The collapsed cone convolution method was used as the radiation dose calculation algorithm. Dose and frequency were determined by the treating radiation oncologist based on risk organs and other factors. The median total irradiation dose was 50 Gy in five fractions (from 40 Gy in four fractions to 56 Gy in eight fractions). The prescription point was D95 (dose covering 95% volume within the PTV). The biologically effective dose (BED) (*α*/*β* = 10 Gy) was 60–100 Gy (median: 100 Gy) (Table 1). The following formula for BED (Gy10) (BED10) was used: BED (Gy10) = *nd* × (1 + *d*/10). The normal liver volume was calculated as the total liver volume minus the gross tumour volume.

### Follow-up

Patients were examined every 1–3 months for 1 year after liver SBRT and thrice monthly thereafter. Laboratory tests were performed at each visit. Treatment responses and intrahepatic recurrences were evaluated according to the clinical practice guideline based on Kimura’s report that the effectiveness of radiotherapy is assessed by dynamic CT/MRI and the local control (LC) is defined as no increase in the size of the treated lesion or early darkening for a period of ≥6 months [26]. Adverse effects were assessed using the Common Terminology Criteria for Adverse Events version 5. Worsening liver reserve was defined as an increase in the mALBI grade or CP scores within 1 year of completing liver SBRT and was the second endpoint of this analysis. Cases of worsening liver reserve that were considered to be due to clear HCC exacerbation (intrahepatic lesions ≥5) were excluded.

### Statistical analysis

Survival rates were calculated using the Kaplan–Meier analysis. A logistic regression model was used for univariate or multivariate analyses to assess the worsening liver reserve after SBRT. Variables used in the multivariate analysis were limited to those with *P*-values of <0.2 in the univariate analysis. Statistical significance was set at *P* < 0.05. Data were analysed using the GraphPad Prism 9 software.

## RESULTS

### Eligible patients

The median follow-up duration was 17.4 months (range, 3.1–82.8 months) for the surviving patients. SBRT was performed as scheduled and feasible for all patients. At the last follow-up, 34/42 (81%) had survived and 8/42 (19%) were deceased.

### Treatment outcomes

**Fig. 1:**
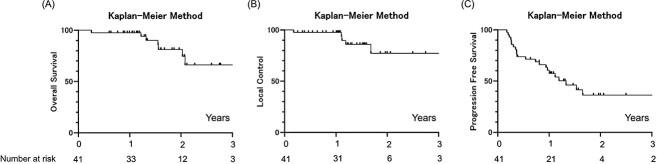
Kaplan–Meier analysis of survival after SBRT for HCC. (**A**) OS curves. (**B**) LC curves. (**C**) PFS curves.

The first local effect was non-progressive disease in 41/42 (98%) and was progressive disease in 1/42 (2%). At censoring during the follow-up, 5/42 (12%) had local progression and 37/42 (88%) did not have local progression. The 1-year overall survival (OS), LC and progression-free survival (PFS) were 100, 98 and 62%, respectively (Fig. 1).

### Toxicity and worsening liver reserve

**Fig. 2:**
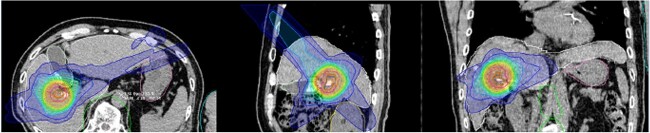
Dose distribution map in axial, sagittal and coronal views.

All liver SBRT procedures were completed without toxicity during the RT period. No acute or Subacute Grade 3 or higher adverse effects were observed. Grade 3 hepatobiliary disorders (repeated cholangitis and biliary bleeding associated with biliary strictures) occurred in only one patient during the chronic phase. This case study targeted the hepatic hilum (Fig. 2). No significant (≥Grade 3) liver enzyme elevation was observed during treatment. Two patients had a history of prior irradiation 2 years after proton treatment but no apparent adverse events.

**Fig. 3:**
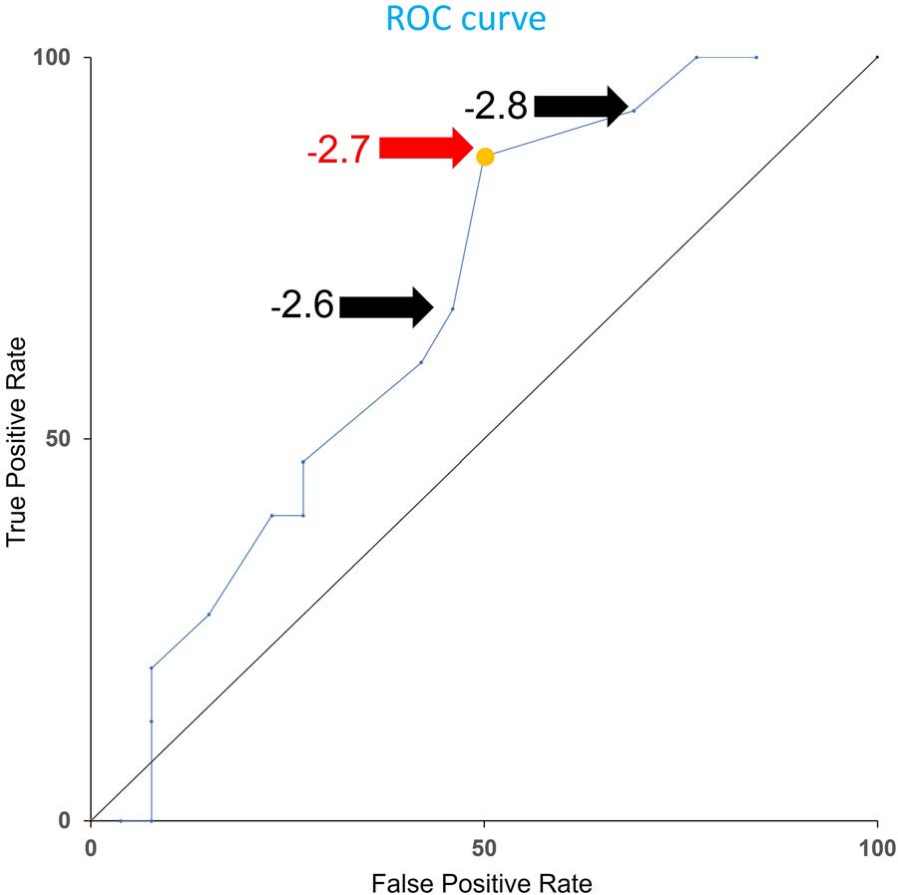
Receiver operative characteristic curve for the ALBI score.

Sixteen of the 42 patients (38%) had worsening liver reserves. The breakdown of worsening liver reserve was as follows: 10 patients had an increase in both mALBI grade and CP score, 3 patients had an increase in mALBI grade only and 3 patients had an increase in CP score only. Of these, there was only one case of worsening liver reserve that was clearly due to exacerbation of HCC, and this case was excluded, and 15/41 (37%) were analysed. From a clinical perspective, the predictor variables included age, sex, ECOG score, CP score, ALBI score, clinical stage, number of lesions, hilar lesions, prior treatment (TACE, RFA or resection), BED, PTV, mean liver dose and normal liver volume. The cut-off value of −2.7 for the ALBI score was calculated as the optimal value based on the receiver operating characteristic curve (Fig. 3). The univariate analyses are summarized in Table 2. The ALBI score was the only significant predictor in the univariate analysis. The final three variables selected for multivariate analysis were age, ALBI score and PTV. Multivariate analysis showed that the ALBI score was the only predictor of worsening liver reserve that was significantly different (Table 3).

**Table 2:** Univariate analysis about predictor variables for worsening liver reserve

	Patients	Reduced liver reserve		*P*-value	RR	95% CI	
	(*n*)	No	Yes				
Age				0.187	2.4	0.6654~9.227	
<80	22	16	6				
≧80	19	10	9				
Sex				0.5992	0.6869	0.1551~2.695	
Male	28	17	11				
Female	13	9	4				
ECOG scale				0.5992	0.6869	0.1551~2.695	
0	28	17	11				
≧1	13	9	4				
CP score				0.6899	1.786	0.06717~47.52	
<7	39	26	14				
≧7	2	1	1				
CP score				0.3117	1.969	0.5278~7.501	
<6	26	18	8				
≧6	15	8	7				
ALBI score				0.0286	6.5	1.422~47.11	
<−2.7	15	13	2				
≧−2.7	26	13	13				
Clinical stage				0.5371	0.6667	0.1770~2.394	
I	22	13	9				
II	19	13	6				
Number of lesions				0.3205	0.4959	0.1131~1.896	
1	26	15	11				
≧2	15	11	4				
Hilar lesion				0.4652	1.917	0.3132~11.81	
Yes	6	3	3				
No	35	23	12				
Prior TACE				0.7811	0.8182	0.1829~3.279	
Yes	29	18	11				
No	12	8	4				
Prior RFA				0.8658	1.179	0.1415~8.029	
Yes	5	3	2				
No	36	23	13				
Prior resection				0.8581	0.8462	0.1066~4.987	
Yes	6	4	2				
No	35	22	13				
BED 100				0.2448	2.159	0.5940~8.164	
Yes	24	14	7				
No	17	9	8				
PTV				0.0978	3.208	0.8501~14.09	
<20 cc	18	14	4				
≧20 cc	23	12	11				
Mean liver dose				0.3948	0.5714	0.1511~2.049	
<8 Gy	21	12	9				
≧8 Gy	20	14	6				
Normal liver volume				0.8371	0.875	0.24~3.143	
<1100 cc	21	13	8				
≧1100 cc	20	13	7				

**Table 3:** Multivariate logistic regression analysis for hepatotoxicity

	Z-value	*P*-value	RR	95% CI
Age	1.153	0.2489	2.391	0.5540 ~ 11.27
ALBI score	2.254	0.0242	7.85	1.552 ~ 63.57
PTV	1.466	0.1426	3.134	0.7119 ~ 15.93

## DISCUSSION

The OS of 100% in this study at 1 year after liver SBRT may be considered as satisfactory, considering that the patient group included older adults (median age, 79 years). The 1-year LC was also very satisfactory at 98%. This study included 38% of cases with a BED10 <100, but there were no significant differences between the LC rates associated with BED10 values of 80 and 100 at least at 1 year. This result is consistent with reports of no difference in the 2-year OS and LC rates for BED10 values <100 and those = 100 [27]. The 1-year OS and LC rates and PFS or intratherapeutic-free survival data after SBRT for HCC from previous reports and the current study are summarized in Table 4 [28–31]. The 1-year OS and LC rates after SBRT were comparable. While the OS and LC were good in these studies, the PFS and intratherapeutic-free survival were generally low.

**Table 4:** Previous and current studies on survival after SBRT for HCC

Study, year	n	CP-B	Dose	BED (Gy10)	1-year OS	1-year LC	1-year PFS or IFS
Andolino, 2011	60	40%	30-48 Gy/3fr	60–71	82%	>90%	70%
Yoon, 2013	93	26%	45 Gy/3-4fr	96–113	86%	95%	52%
Takeda, 2014	63	16%	35-40 Gy/5fr	60–72	100%	100%	76%
Huertas, 2015	77	14%	45 Gy/3fr	113	82%	99%	69%
Current study	42	2%	40–56 Gy/4-8fr	80–100	100%	98%	62%

In this study, the proportion of cases with CP Grade A was relatively high at 95%, and there were no RILDs above Grade 3. This is consistent with previous reports that CP Grade B is associated with a higher frequency of RILD of Grade 3 or higher [11–13]. However, non-severe cases should also be evaluated because HCC is associated with a high risk of recurrence and liver dysfunction after RT, which is relevant to future management. This makes sense, especially for cases such as those in this study, where the majority had CP Grade A. In fact, 15/41 (37%) of the patients in this study had a worsening liver reserve. There was a significant difference in the liver reserve, but this was reflected in the baseline ALBI but not in the CP score. This finding is consistent with previous reports that the ALBI score is useful for assessing the hepatotoxicity after SBRT for HCC [17–19]. The liver reserve was deteriorated in two patients in the good group with ALBI scores of <−2.7. The only Grade 3 adverse event in this study occurred in one of these cases, in which the target was the hepatic hilum. The hepatic hilum is located adjacent to the major blood vessels (inferior vena cava, primary hepatic veins and portal vein) and common bile duct. The anatomical structures located in this region are complex, making surgical resection or RFA difficult and hazardous [32, 33]. Therefore, SBRT is a promising alternative treatment for hepatic hilar tumours [34]. Even with SBRT, hepatic hilar lesions remain anatomically vulnerable to adverse events. Hepatotoxicity (defined as a CP score worsening ≥2) was observed in 7/41 (17%), which were too few to analyse.

This study has some limitations. The sample was too small for multivariate analysis using all items of potential clinical significance; therefore, a stepwise method was used to narrow down the variables. Our definition of worsening liver reserve included worsening CP score or mALBI grade, but it is not clear whether CP score or mALBI grade alone should be evaluated or which is better. Because both are currently used in practice to assess liver reserve, we included both as outcomes in this study. There were a certain number of patients who experienced only one exacerbation each, which is believed to be due to the fact that mALBI grade and CP score are discontinuous variables and thus have different thresholds. In this sense, it may be better to look at the outcomes in terms of worsening of the ALBI score itself, which is a continuous variable, but there is still no indicator to determine the number of points above which an increase is considered worsening. It is also unclear whether our definition of worsening liver reserve was restricted by SBRT. However, there was no apparent significant difference in the liver reserve when analysed according to pre-treatment (TACE, RFA and resection), although this study was limited to SBRT cases, and we excluded a case of worsening liver reserve associated with an apparent exacerbation of HCC.

## CONCLUSION

We found that the ALBI score was the only significant predictor for worsening liver reserve after SBRT in elderly Japanese patients. Therefore, the baseline ALBI score could be a useful risk predictor for liver toxicity after SBRT.

## PREVIOUS PRESENTATION

This manuscript has not been published or presented elsewhere in part or entirety and is not under consideration by another journal.
